# Magnetic Ordering in Sr_3_YCo_4_O_10+x_

**DOI:** 10.1038/srep19762

**Published:** 2016-01-28

**Authors:** Takayoshi Kishida, Myron D. Kapetanakis, Jiaqiang Yan, Brian C. Sales, Sokrates T. Pantelides, Stephen J. Pennycook, Matthew F. Chisholm

**Affiliations:** 1 Department of Technology Group, Analysis & Simulation Center, Asahi Kasei Corporation, 2-1 Samejima, Fuji, Shizuoka 416-8501, Japan; 2 Department of Physics and Astronomy, Vanderbilt University, Nashville, Tennessee 37235, USA; 3 Materials Science and Technology Division, Oak Ridge National Laboratory, Oak Ridge, Tennessee 37831, USA; 4 Department of Materials Science and Engineering, National University of Singapore, Singapore 117576 Singapore

## Abstract

Transition-metal oxides often exhibit complex magnetic behavior due to the strong interplay between atomic-structure, electronic and magnetic degrees of freedom. Cobaltates, especially, exhibit complex behavior because of cobalt’s ability to adopt various valence and spin state configurations. The case of the oxygen-deficient perovskite Sr_3_YCo_4_O_10+x_ (SYCO) has attracted considerable attention because of persisting uncertainties about its structure and the origin of the observed room temperature ferromagnetism. Here we report a combined investigation of SYCO using aberration-corrected scanning transmission electron microscopy and density functional theory calculations. Guided by theoretical results on Co-O distances projected on different planes, the atomic-scale images of several different orientations, especially of the fully oxygenated planes, allow the unambiguous extraction of the underlying structure. The calculated magnetic properties of the new structure are in excellent agreement with the experimental data.

The oxygen-deficient perovskite Sr_3_YCo_4_O_10+x_^1^ exhibits the highest ferromagnetic ordering temperature of any of the perovskite cobaltates with T_c_ = 335K^2^. There have been several contending structural models for SYCO, extracted from X-ray and neutron diffraction, but there is no consensus on the precise atomic structure of this material^3^,^4^,^5^,^6^. The undisputed facts are that SYCO exhibits antiferromagnetic ordering with asymmetric spin-up and spin-down magnetic moments, characteristic of ferrimagnetism. The different structural models correspond to different origins for the observed ferrimagnetism, with no apparent resolution.

Atomic resolution imaging by scanning transmission electron microscopy (STEM) can now be routinely performed using the high-angle annular dark field (HAADF) mode of imaging. Recently, an analysis using atomic column shapes obtained by HAADF-STEM imaging was found to be a sensitive method to detect atomic distortions and octahedral tilts induced by oxygen atoms as reported by Borisevich *et al.*^7^. The STEM annular bright field (ABF) imaging technique, combined with aberration correction, is another relatively new technique that can reveal the positions of lighter atom columns, in addition to the heavier atom columns^8^,^9^,^10^,^11^.

In this article, we report a new structure model of SYCO constructed using primarily ABF-STEM images and column shape analysis. By comparing with previously reported models it becomes clear that our new model is in excellent agreement with the experiment. Acompanied by theoretical calclulations based on the density functional theory (DFT) the model is used to study the origin of the magnetism on SYCO.

## Results and Discussion

**Figure 1:**
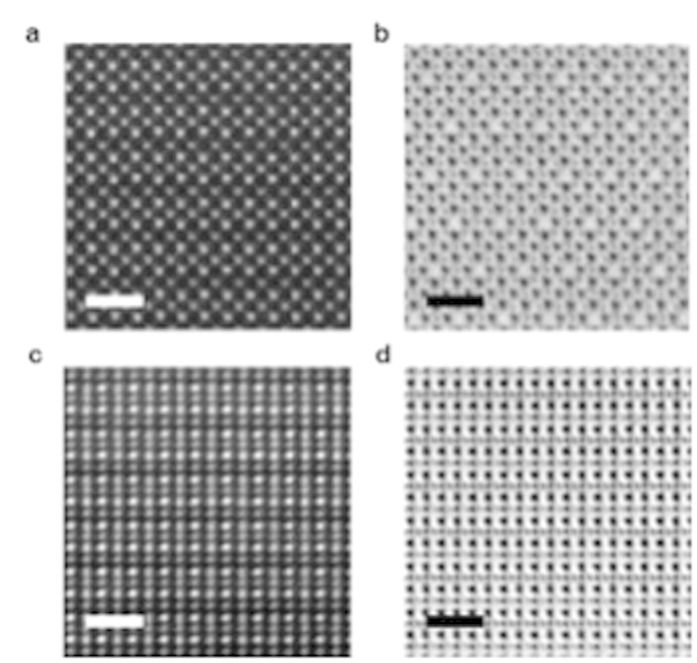
Single-domain, atomically resolved STEM images of SYCO. **(a)** annular dark-field and **(b)** annular bright-field images along a 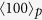. **(c)** annular dark-field and **(d)** annular bright-field images of SYCO along a 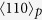 direction. Scale bar is 1 nm.

**Figure 2:**
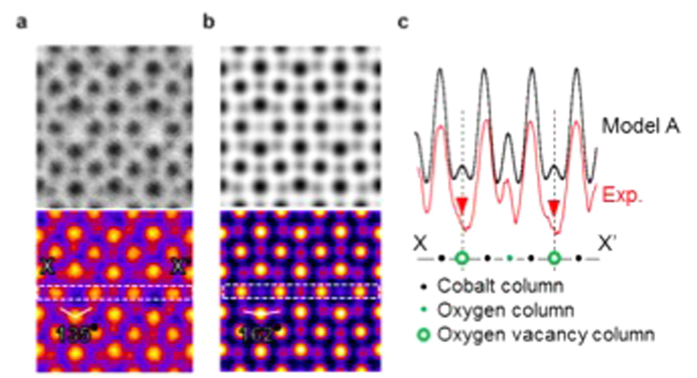
ABF-STEM images viewed along a 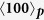 direction. (**a**) Experimental annular bright-field image of SYCO viewed along the [100]_*p*_ direction. O-Sr/Y-O angle is 135˚. **(b)** Simulated annular bright-field images using a crystal structure proposed by Sheptyakov *et al.* O-Sr/Y-O angle is 162˚. (**c)** Intensity profiles across the dashed rectangle through the Co-O plane containing oxygen vacancy columns.

Figure 1a,b shows atomic resolution ADF-STEM and ABF-STEM images viewed along a <100>_*p*_ direction of a unit cell based on a pseudo-cubic perovskite structure. Figure 1c,d show views along a <110>_*p*_ direction. These images are the result of summing ten sequentially acquired frames after alignment by cross-correlation^12^, with no other image processing being applied. These images are from thin, single domain regions of the specimens (Supplementary Figure S1 containing lower magnification images show that the crushed powder SYCO specimens contain at least three, orthogonal domains). Figure 2 compares the experimental ABF-STEM image along a <100>_*p*_ direction to a simulated image from the model proposed by Sheptyakov *et al.*^3^ (model A). Other proposed models (Nakao *et al.*^4^ (x = 0.5), Khalyavin *et al.*^5^ (x = 1), and Bettis *et al.*^6^ (x = 1), which we refer to as model B, C and D respectively) show practically identical simulated images (see Supplementary Figure S2). As neither Khalyavin *et al.*^5^ nor Bettis *et al.*^6^ reported details of the oxygen vacancy ordering, we prepared a model B2 and C2 including a zig-zag oxygen vacancy ordering structure as noted in refs 5,6 respectively. In the lower panels the image contrast is inverted and colored to more clearly show the intensity variation present at the oxygen ‘vacancy’ column position. All models differ from the experimental <100>_*p*_ data in the following two aspects: First, the experimental intensity of the oxygen vacancy columns is very weak or absent, as seen in the line profile of Fig. 2c extracted from the white dashed square indicated in Fig. 2a,b. In contrast, the O vacancy columns in the simulated image of model A have clear peaks. These results imply that the oxygen vacancy columns within the oxygen-deficient layer have a lower oxygen concentration and/or different oxygen distribution compared to the previously proposed models. Second, the positions of the oxygen columns in the horizontal Sr-O planes, indicated by white arrows in the image colored in Fig. 2a are shifted toward the oxygen vacancy columns compared with the simulated results based on previous models. In the experimental image, the O-Sr/Y-O angle is 135˚, significantly smaller than the angles present in all previous models, which are 162˚, 156˚, 160˚ and 159˚ respectively for model A, B, C and D (see Supplementary Figure S2).

**Figure 3:**
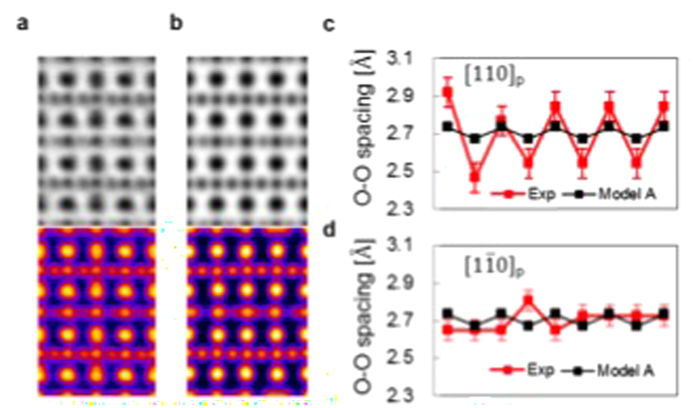
ABF images viewed along a 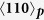 direction and O-O spacings. **(a)** Upper panel is experimental annular bright-field image as viewed along [110]_*p*_ direction. Lower panel show the inverted contrast and colored version of the image in the upper panel. **(b)** Upper panel shows a simulated annular bright-field image based on Model A proposed by Sheptyakov *et al.* as viewed along 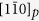 direction. Lower panel shows the inverted contrast and colored version of the image in the upper panel. **(c)** O-O spacing as viewed in the [110]_*p*_ projection of the fully oxygenated Co-O layer. d) O-O spacing as viewed in the 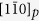 projection of the fully oxygenated Co-O layer.

Figure 3 compares the experimental ABF-STEM image (Fig. 3a) along a <110>_*p*_ direction to a simulated image of the structure proposed by Sheptyakov *et al.*^3^ (model A Fig. 3b). We observe a shift of the oxygen column positions, relative to the heavier cation columns, which is due to the specimen mis-tilt or because the detector is non-centered. This apparent shift has been discussed in detail by Findlay *et al.* in ref. 13. However, what we measure in our work is the oxygen-oxygen separation and because these oxygen columns have similar environments their electron channeling effects should also be similar and, thus, the shifts of the features corresponding to the oxygen columns are constant. Thus, detector offsets can produce relative shifts between cobalt (and Sr) column features and oxygen column features, but distances between oxygen column features in this fully oxygenated plane are not affected by a non-centered detector. Although such shifts become apparent for the light oxygen atoms the O-O spacings are not affected since the involved oxygen atoms have similar enviroment. Figure 3c,d show O-O spacings within the fully oxygenated Co-O plane. We found two different spacing distributions in the experimental images revealing that the orthogonal <110>_*p*_ projections are different. As shown in Fig. 3c, the experimentally projected O-O spacing seen in a [110]_*p*_ view of SYCO alternates between 2.85 ± 0.05 *Å* and 2.53 ± 0.03 *Å*. When viewed along the 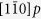 direction, the experimentally projected O-O spacing in the fully oxygenated layer is between 2.68 ± 0.04 *Å* and 2.73 ± 0.06 *Å* with no obvious periodic variation. The images indicate a 2 × 1 variation in the O-O spacing, which we will show directly reflects the variation of the Co spin state in the fully oxygenated CoO_6_ plane.

**Figure 4:**
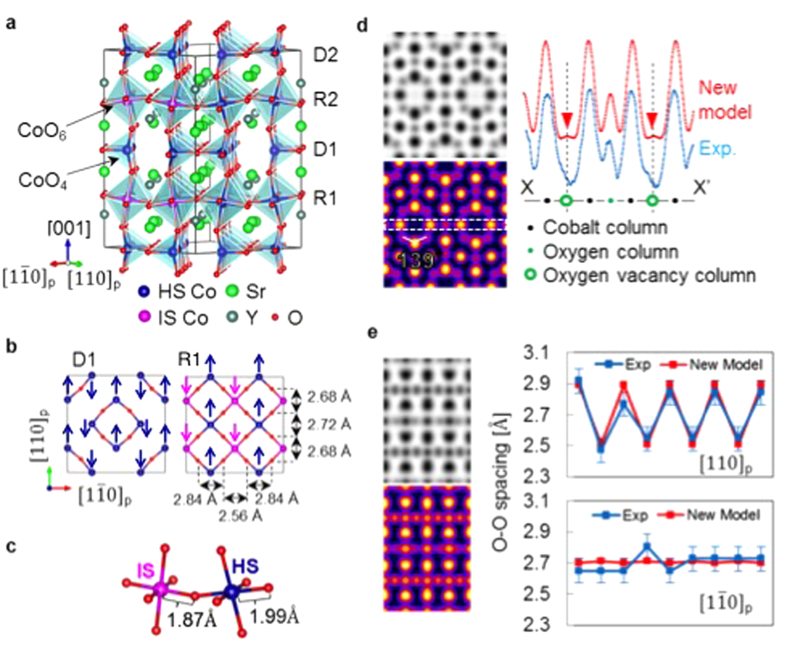
The magnetic structure of SYCO. **(a)** Illustration of the new crystal structure of SYCO with fully oxygenated (R1-R2) and oxygen deficient (D1-D2) layers. **(b)** The oxygen deficient layer (D1) and the fully oxygenated layer (R1) as viewed along the [001] direction, the arrows show the direction of the magnetization for each cobalt atom. In the fully oxygenated layers, columns with intermediate spin (−1.65 *μ*_*B*_) along the [110]_*p*_ and 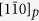 directions are observed. The distances displayed are the projected O-O spacing viewed along 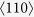 directions, obtained from atomic structure model. **(c)** Schematic of the Co-O bonds associated with the alternating high spin state (HS) and intermediate spin state (IS) cobalt. **(d)** Simulated annular bright-field [100]_*p*_ images using the new model with intensity profile across the dashed rectangle through the oxygen-deficient Co-O plane. **(e)** Simulated annular bright-field 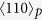 image using the new model along with O-O spacing within the fully oxygenated Co-O layer.

To better match the experimental images, a new model based on the Sr_3_YCo_4_O_10.76_ structure reported by Sheptyakov *et al.* was constructed but with a lower O concentration (x = 0)^3^. In the new model, the oxygen atoms identified as O2 and O2′, as in ref. 5, were removed and the oxygen O3 site was shifted to better match our images. Our DFT calculations indicate that the lowest energy structure displays a G-type antiferromagnetic spin ordering of the Co ions in agreement with previous measurements^2^. The calculated structure based on a tetragonal structure (I4/mmm) is orthorhombic (F/mmm) with lattice parameters and atom positions listed in Supplementary Table S1. Our x-ray diffraction measurements shown in Figure S3 of the Supporting Information are consistent with the F/mmm space group. The calculations indicate that within the oxygen-deficient layer the cobalt magnetic moments are anti-ferromagnetically ordered as shown in Fig. 4b with a magnitude of about 2.7μ_B_ per Co atom. More interesting is the spin ordering in the fully oxygenated layers where the formation of columns with intermediate spin (−1.65 μ_B_) along the 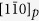 and 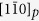 directions are observed, as shown in Fig. 4b. The intermediate spin (IS) columns alternate with high spin (HS) columns of magnitude about 2.54μ_B_ per cobalt atom. This results in a calculated net magnetization of 0.22μ_B_ per cobalt atom, which is consistent with the measurement of the magnetic susceptibility as a function of the temperature (see Figure S4 in the Supporting Information). This calculated spin structure of SYCO is different from those of previously published models including that of Sheptyakov *et al.* (ref. 3) who also predicted ferrimagnetism but as the result of different spins on the two different Co-O layers. Moreover, variations in spin states are directly reflected in the crystal structure. The fully oxygenated layers have two different types of CoO_6_ octahedra, in which the calculated in-plane Co-O bond length (1.87 Å) of every IS Co atom is smaller than the corresponding bonds (1.99 Å) of the HS Co atoms (Fig. 4d). The IS/HS picture obtained in these calculations was reproduced in a larger cell built by expanding the original one by a factor of two along the [110] and 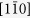 directions. This indicates that the smaller supercell was large enough to reflect the spin structure.

Image simulations (ABF-STEM) using the new model are shown in Fig. 4d. In the <100>_*p*_ view, the O-Sr/Y-O angle for the new model is 139 degrees, in good agreement with the experimental image (Fig. 2a). The new model also has much weaker O vacancy column intensity, as shown in Fig. 4d, again in much better agreement with experiment. Furthermore, the new model produces a much better match with the experimental ABF-STEM images along [110]_*p*_ and 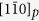 directions. As described in the Supplementary Figure S5 and S6, the shapes of the Sr/Y columns and Sr/O columns seen in the experimental images better match those obtained from the new model than those from previous models. These shapes originate from atomic arrangements along the columns that are too small to be resolved (as separated features) but are large enough to make the feature non-circular. The most important confirmation of the model structure is seen in the in-plane Co-O bond lengths within the fully oxygenated CoO_6_ plane. Our calculated structure indicates that the in-plane Co-O distance is 1.87 Å for IS Co and 1.99 Å for HS Co (Fig. 4c). When viewed along the [110]_*p*_ and 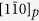 directions, the Co columns in the fully oxygenated layer of our model structure are predicted to alternate between the HS and IS state (Fig. 4b,c). The result of this is that the projected O-O distance when viewed in the [110]_*p*_ direction should alternate between 2.84 Å when a HS Co is located between the two O columns and 2.56 Å when an IS Co is located between the O columns (see Fig. 4b). When viewed along the  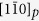 direction the predicted O-O distance is 2.72 Å when bracketing HS Co and 2.68 Å when bracketing IS Co. Measured O-O distances can therefore directly reveal the Co spin states in the experimental images. Indeed, the calculated location and periodicity of the O columns in our model, as shown in Fig. 4e, reproduce those seen in the actual structure, strongly supporting our calculated spin state arrangement. The new model is the only one to reproduce all the experimental ABF-STEM image feature shapes, intensities, distortions and angles.

Although our proposed new model is a better fit to experiment than the other proposed crystal structures, the oxygen concentration of our model is Sr_3_YCo_4_O_10_, which is lower than that expected for oxidized powder. Therefore, further refinement will be needed to include the additional oxygen atoms within the oxygen-deficient layer. Given the good fit with our basic model, the additional O atoms most likely do not occupy well-ordered positions. The additional O atoms appear to be distributed over a variety of possible sites that do not give rise to extra intensity nor do they change the basic column shapes revealed in our images.

In conclusion, we propose a new model for SYCO constructed using aberration-corrected Z-contrast and annular bright field imaging combined with DFT calculations. The model gives much improved agreement with experimental images. The calculated relaxed structure model obtained from these images has antiferromagnetic Co^3+^ ions in the high spin state in the oxygen-deficient tetrahedral CoO_4.25_ layers. Our model and images show that the observed ferromagnetism in SYCO arises from the fully oxygenated CoO_6_ octahedral layers. The Co^3+^ ions in these layers are antiferromagnetically aligned with alternating high spin and intermediate spin resulting in ferrimagnetism. Importantly, we find, in the ABF-STEM images, periodic variations in Co-O bond lengths in the fully oxygenated Co-O layers arising from these alternating high spin and intermediate spin Co atoms.

## Methods

### Synthesis and sample preparation

The SYCO sample for the study was synthesized by a solid-state reaction process as reported in ref. 2. Magnetic properties were measured with a Quantum Design (QD) magnetic properties measurement system in the temperature interval 2 *K* ≤ *T* ≤ 400  *K*, using a field-cooling mode in an applied magnetic field of 500 Oe. The measurements confirmed that the sample used in this study exhibits ferromagnetism below 335 *K* (see Figure S3 in the Supporting Information) with a net moment of about 0.25 *μ*_*B*_ per Co as reported in ref. 2. Specimens for STEM were prepared by crushing SYCO powder to avoid any surface damage and any structural change due to ion milling.

### STEM imaging

The atomic resolution ADF- and ABF-STEM images were acquired with an aberration corrected Nion UltraSTEM 200, operated at 200 kV. STEM-ABF image simulations were carried out using the Multi-slice code provided by HREM Inc. assuming a probe-forming aperture half angle of 30mrad and half angles of the ABF detector spanning 15 to 30 mrad.

### First-principles calculations

All numerical calculations were performed within the framework of density function theory (DFT) as implemented in the Vienna ab initio simulation Package (VASP)^14^,^15^,^16^ using a 7.64 Å × 7.64 Å × 15.32 Å computational cell that contains 72 atoms in total. The convergence of the numerical results was further confirmed by calculations on a larger cell built by expanding the original one by a factor of two along the [110] and 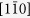 directions. In order to take into account the correlation effects of the 3d electrons of Co ions we employ the GGA + U approximation with a typical value of the effective Hubbard parameter of U = 3eV using the Dudarev approach and an energy cut-off of 600eV for the plane wave basis set^17^. In order to ensure the convergence of the density of states we sampled the Brillouin Zone using a 4 × 4 × 4 k-point mesh. The ionic relaxation of the structure was performed by assuming various spin arrangements as the initial configuration of the cobalt atoms magnetic moments^18^.

## Additional Information

**How to cite this article**: Kishida, T. *et al.* Magnetic Ordering in Sr_3_YCo_4_O_10+x_. *Sci. Rep.* **6**, 19762; doi: 10.1038/srep19762 (2016).

## Supplementary Material

Supplementary Information
