# Supplementary material for: Magnetic Ordering in Sr3YCo4O10+x
Source: Sci Rep. 2016 Jan 28;6:19762. doi: 10.1038/srep19762 (PMC4730147; doi:10.1038/srep19762)
Supplement: Supplementary Information [file srep19762-s1.pdf]

Supporting Information

Magnetic Ordering in $Sr_3YCo_4O_{10+x}$

Takayoshi Kishida[1], Myron D. Kapetanakis[2,3,*], Jiaqiang Yan[3], Brian C. Sales[3], Sokrates T. Pantelides[2,3], Stephen J. Pennycook[4], Matthew F. Chisholm[3,*]

[1]Department of Technology Group, Analysis & Simulation Center, Asahi Kasei Corporation, 2-1 Samejima, Fuji, Shizuoka 416-8501, Japan.
[2]Department of Physics and Astronomy, Vanderbilt University, Nashville, Tennessee 37235, USA.
[3]Materials Science and Technology Division, Oak Ridge National Laboratory, Oak Ridge, Tennessee 37831, USA.
[4]Department of Materials Science and Engineering, National University of Singapore, Singapore 117576 Singapore.

*Correspondence and requests for materials should be addressed to M.D.K. (email: myron.kapetanakis@Vanderbilt.Edu) and M. F. C. (chisholmmf@ornl.gov)

This file includes: Supplementary figures S1-S6, Supplementary Table S1.

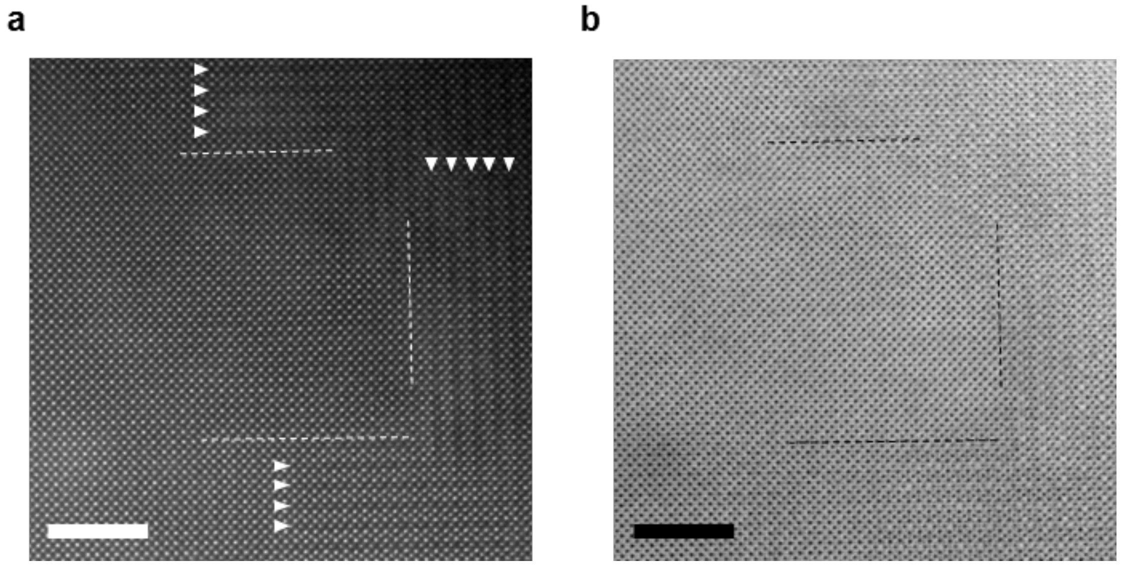

Figure S1. a) Atomic-resolution ADF STEM image of SYCO showing domains with <110>, <1-10> and <001> projections, obtained with detector range 30-200mrad. b) Simultaneously obtained atomic-resolution ABF STEM image with detector range 15-30mrad. Oxygen-deficient layers are arrowed, revealing the domain structure. Scale bar is 4nm.

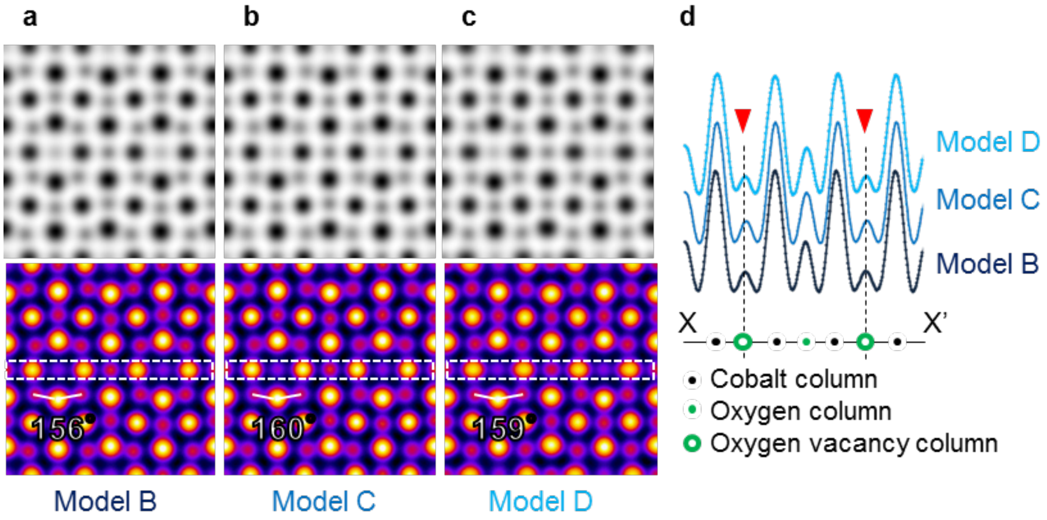

Figure S2. a-c) Simulated ABF STEM images using the crystal model B proposed by Nakao et al., the crystal model C proposed by Khalyavin et al., and the crystal model D proposed by Bettis et al.

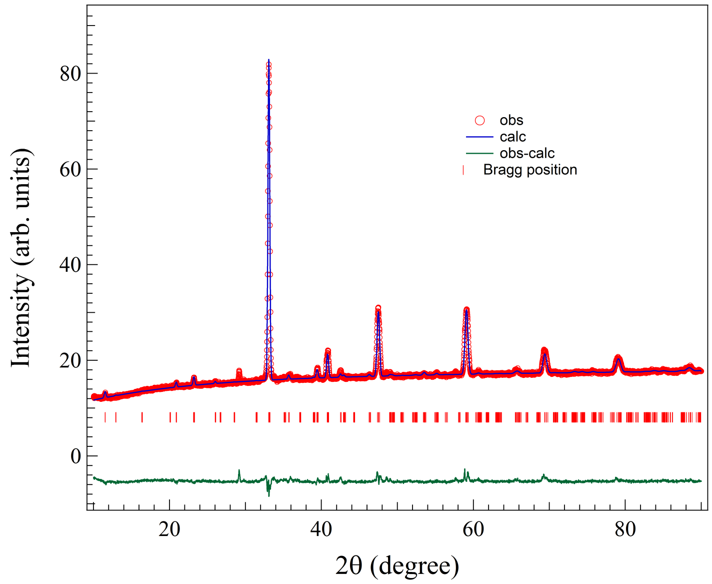

Figure S3. Fullproof refinement of room temperature x-ray powder diffraction using the F/mmm structural model proposed in this work. The peak around 30 degree is from Y_2O_3 impurity. The pattern obtained by the calculated structure (solid blue line) is in good agreement with the experimentally observed one (open circles). Patterns obtained by other crystal models match the calculated pattern based on our model and are not shown here. We are unable to distinguish which structural model provides the best fit the diffraction pattern. Our model can index the reflections, both nuclear and magnetic. Only single domain diffraction studies could provide data to confirm the detailed structure distortions we measure by STEM.

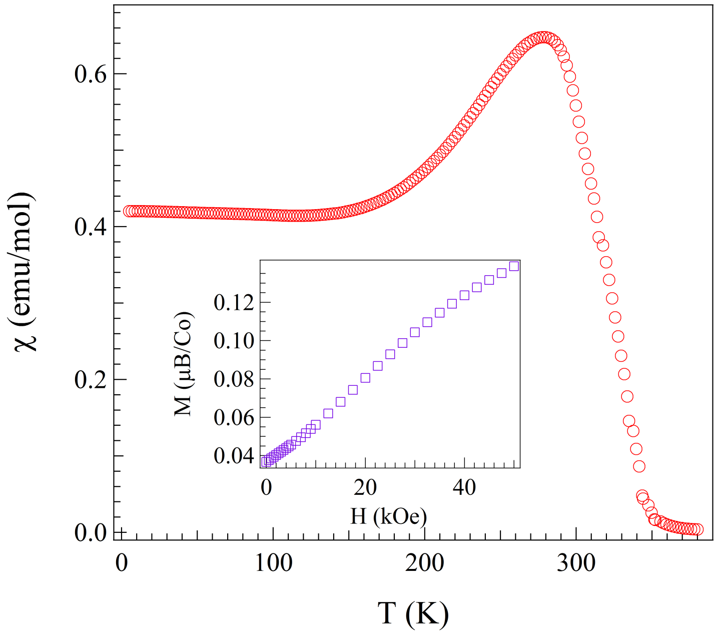

Figure S4. The temperature dependence of magnetic susceptibility measured in an applied magnetic field of 500 Oe. The system remains ferromagnetic for temperatures up to about 335 K. Inset shows the field dependence of magnetization at 2 K.

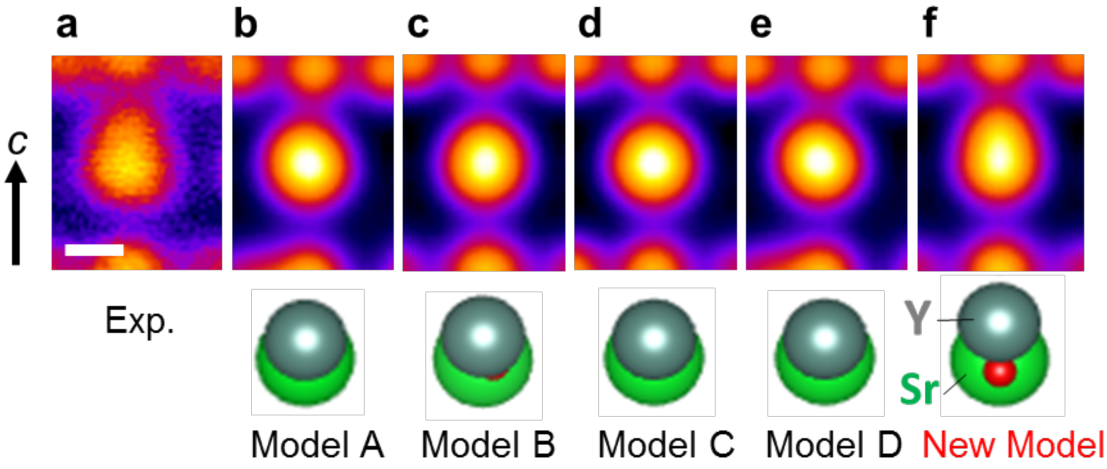

Figure S5. Shape of the Sr/Y column viewed along <110>$_p$ direction. a) the shape of the Sr/Y column seen in experimental ABF STEM image. b-f) The shape of the Sr/Y column seen in simulated ABF STEM images using the crystal model A proposed by Sheptyakov et al., the crystal model B proposed by Nakao et al., the crystal model C proposed by Khalyavin et al., the crystal model D proposed by Bettis et al., and the new crystal model proposed in this letter. It is clear that the shapes of the Sr/Y columns in the experimental image indicated in a are not circles but slightly elongated along the c direction in a pear-like shape. This shape originates from the atomic arrangement where the yttrium atoms are displaced away from the strontium atom positions along the c direction as shown in the sketch of f, rather larger than the shift in model A-D, as shown in the sketch in lower panel of b-e. Scale bar is 1 Å.

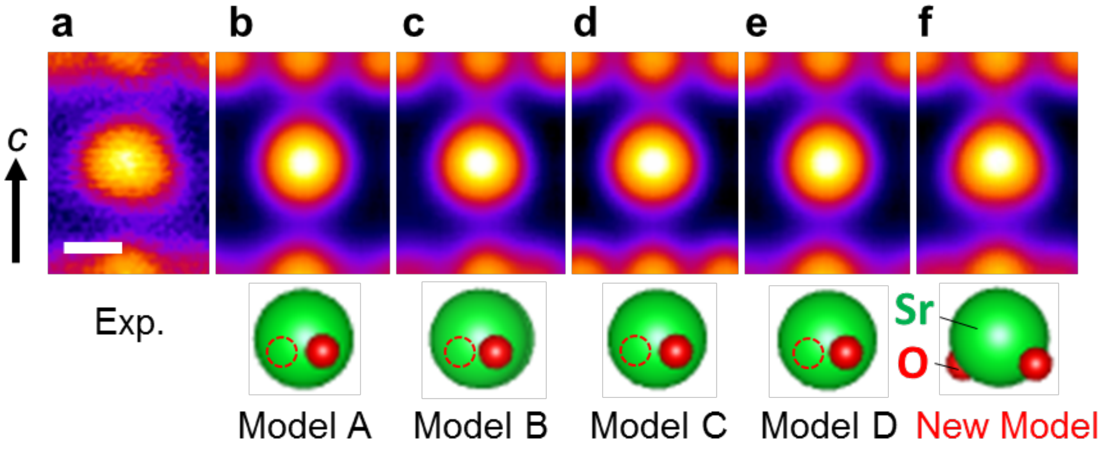

Figure S6. Shape of the Sr/O column viewed along <110>$_p$ direction. a) the shape of the Sr/O column seen in experimental ABF STEM image. b-f) the shape of the Sr/O column seen in simulated ABF STEM images using the crystal model A proposed by Sheptyakov et al., the crystal model B proposed by Nakao et al., the crystal model C proposed by Khalyavin et al., the crystal model D proposed by Bettis et al., and the new crystal model proposed in this letter. The Sr/O column shape in a is seen that they also are not circular but more like a chestnut shape. This shape of the Sr/O column originates because the oxygen atomic positions within the Sr/O columns are displaced away from the Sr atom positions as shown in the sketch in f, rather than the shifts proposed in the models A-D, as shown in the sketch in b-e. Scale bar is 1 Å.

Table S1. Calculated atomic coordinates and magnetic moments M (μ_B) for the new orthorhombic model of SYCO (space group F/mmm).

Atom	x [Å]	y [Å]	z [Å]	M [μ_B]
Y1	0	0	0.1667	0.002
Sr1	0	0	0.61628	-0.001
Sr2	-0.25	0.25	0.13154	-0.002
Co1	0	0.25	0	2.72
Co2	-0.25	0.5	0	-2.74
Co3	0	0.25	0.25	-1.65
Co4	-0.25	0.5	0.25	2.54
O1	0	0.19609	0.10977	0.18
O2	0.30160	0.5	0.11099	-0.193
O3	-0.13688	0.36317	0	-0.003
O4	-0.11862	0.12592	0.26866	0.037 0.043
